# No detectable truncating mutations in large T antigen (LT-Ag) sequence of Merkel cell polyomavirus (MCPyV) DNA obtained from porocarcinomas

**DOI:** 10.1186/s13027-024-00568-5

**Published:** 2024-03-21

**Authors:** Rosaria Arvia, Mauro Sollai, Daniela Massi, Patricia Asensio-Calavia, Carmelo Urso, Krystyna Zakrzewska

**Affiliations:** 1 Department of Experimental and Clinical Medicine, University of Florence, Viale Morgagni 48, 50134 Florence, Italy; 2 Department of Pathology, IRCC Humanitas Research Hospital, Milan, Rozzano, Italy; 3 Division of Pathological Anatomy, Department of Health Sciences, University of Florence, Florence, Italy; 4 Biotechnology of Macromolecules Research Group, Instituto de Productos Naturales y Agrobiología (IPNA-CSIC), San Cristóbal de La Laguna, Spain; 5 Dermatopathology Study Center of Florence, Florence, Italy

**Keywords:** Merkel cell polyomavirus, Porocarcinoma, MCPyV large T antigen, FFPE samples

## Abstract

Merkel cell polyomavirus (MCPyV) is associated with Merkel cell carcinoma (MCC). In tumor cells the MCPyV large T antigen (LT-Ag) is frequently found truncated and this is considered a major tumor-specific signature. The role of MCPyV in other, non-MCC tumours, is little known. Viral DNA and/or tumour-specific mutations have been sometimes detected in different tumours, but such data are not unequivocal and the involvement of the virus in the tumorigenesis is not clear. In a previous study, we demonstrated a significantly higher prevalence of MCPyV DNA in formalin fixed paraffin embedded (FFPE) porocarcinoma tissues compared to the normal skin.

In the present study, we investigated the presence of truncating mutations in MCPyV LT-Ag coding region in porocarcinoma specimens. Using several overlapped PCR primer pairs, the complete LT-Ag sequence from two biopsies were obtained. No truncating mutations were detected.

The lack of truncating mutations in LT-Ag sequence does not seem to support the role of MCPyV in porocarcinoma oncogenesis. However, an oncogenetic mechanism, different from that proposed for MCC and not associated with the LT-Ag mutations/deletions, cannot be excluded. Further studies of more sequences coding for LT-Ag would be needed to verify this hypothesis.

Dear Editor,

Merkel cell polyomavirus (MCPyV) has been classified by World Health Organization’s International Agency for Research on Cancer as a group 2a carcinogen (“probably carcinogenic to humans”). It was due to the preponderance of evidence associating MCPyV with the pathogenesis of Merkel cell carcinoma (MCC), a rare, aggressive skin cancer developing in sun-exposed skin of elderly and immunosuppressed patients [1]. Among viral markers detected in MCPyV-associated MCC, there are high prevalence of viral DNA in tumour biopsies, clonal integration of viral DNA into tumour cell genome and presence of mutations or deletions in the sequence coding for MCPyV Large T antigen (LT-Ag). These mutations lead to a premature truncation in the viral LT helicase, resulting in blockage of MCPyV replication capacity, inhibition of tumour suppressor RB protein and upregulation of E2F transcription factors, triggering the S phase of the cell cycle. The presence of such mutations is considered a major tumor-specific signature [2, 3].

A high prevalence of MCPyV DNA in other skin cancers such as squamous cell carcinoma (SCC), basal cell carcinoma (BCC) or Bowen’s disease (BD), mainly in immunocompromised patients, has been also reported [4–9]. Moreover, tumor-specific mutations of LT-Ag have also been demonstrated in a number of cases of chronic lymphocytic leukemia [10], non-small cell lung cancer [11] and in SSC of the skin [5].

Porocarcinoma is the most frequent adnexal sweat gland carcinoma, characterized by a high mortality rate. Although several cases have been associated with radiotherapy, the aetiology of this tumour is largely unknown. In our previous study, using ddPCR, we demonstrated for the first time a significantly higher prevalence of MCPyV DNA in porocarcinoma specimens, than in skin biopsies obtained from healthy subjects (68% vs. 30%, *p* < 0.04) [12]. This observation prompted us to investigate the presence of truncating mutations in MCPyV LT-Ag coding sequence obtained from porocarcinoma biopsies.

Twenty MCPyV genome sequences downloaded from NCBI (National Centre for Biotechnology Information) database were aligned using ClustalW v1.4 included in BioEdit v7.0.0 to create consensus LT-Ag sequence. Based on this sequence, twelve overlapped primer pairs to amplify entire LT-Ag region were subsequently designed (Table 1).

**Table 1:** Primer pairs used to amplify MCPyV LT-Ag sequence

Name	Sequence
137 F-1122R	Forward 5’ GTGAGGTAGCTCATTTGCTCC 3’ Reverse 5’ CGGGAGGTATATCGGGTCCT 3’
137 F -569R	Forward 5’ GTGAGGTAGCTCATTTGCTCC 3’ Reverse 5’ACAATGCTGGCGAGACAACT 3’
615 F– 1538R	Forward 5’ CTGTCTGACGTGGGGAGAGTG 3’ Reverse 5’TTTTGGCTTTGGTGGAGTGC 3’
615 F– 1122R	Forward 5’ CTGTCTGACGTGGGGAGAGTG 3’ Reverse 5’ CGGGAGGTATATCGGGTCCT 3’
1101 F– 1538R	Forward 5’AGAGGACCCGATATACCTCCC 3’ Reverse 5’ TTTTGGCTTTGGTGGAGTGC 3’
1290 F– 2085R	Forward 5’ GAGCCTCCCTCGTCCTCTGAGG 3’ Reverse 5’CCTTGTGAGGTTTGAGGCGAGAT 3’
1482 F– 2085R	Forward 5’ AGCTCTGCAAGCTCTGCTAGT 3’ Reverse 5’ CCTTGTGAGGTTTGAGGCGAGAT 3’
1793 F– 2209R	Forward 5’ TACCTTCTGCACTATAAGCTTT 3’ Reverse 5’CTGGTCATTTCCAGCATCTCT 3’
1935 F– 2652R	Forward 5’ GAGGCCAGCTGTAATTGGAATT 3’ Reverse 5’AGCTTACAGCTACAGCACCG 3’
1935 F– 2518R	Forward 5’ GAGGCCAGCTGTAATTGGAATT 3’ Reverse 5’GCACACCCTAGTTCAAAAGGC 3’
2577 F– 3003R	Forward 5’ GATCTGCAACCAGGGCAAGGAAT 3’ Reverse 5’GAGGGTCCTGACCAGCTTCTA 3’
2577 F– 3204R	Forward 5’ GATCTGCAACCAGGGCAAGGAAT 3’ Reverse 5’AGGACCTCAAAAGGCCTCTCA 3’

Position of LT gene primers is referred to reference sequence Merkel cell polyomavirus isolate HF (access number JF813003).

MCPyV positive FFPE tissue samples were already collected in previous investigation [12] after local Ethics Committee approval.

Total DNA was extracted from 9 porocarcinomas, from two salivary swabs (containing replication-competent virus) and from two MCC biopsies (presumably containing MCC-specific truncating mutation, as reported in the literature).

DNA from tissue samples was extracted from 4 to 5 freshly cut sections of formalin fixed paraffin embedded (FFPE) specimens, 10 μm thick using QIAamp DNA FFPE Tissue kit, (Qiagen Valencia, CA, USA), while DNA from salivary swabs was extracted using High Pure PCR Template Preparation Kit (Roche), according to the manufacturer’s instructions. The PCR amplifications were performed in 25 µl of reaction mix containing 12.5 µl of SsoAdv Universal SYBR Green PCR Super Mix 2x (Bio-Rad), 1 µl of primers mix (20 µM) and 3 µl of extracted DNA. The PCR amplification was carried out on Rotor Gene 6000 (Qiagen Valencia, CA, USA) with the following thermal profile: hold at 95 °C for 15 min, 40 cycles of 94 °C for 15 s, 60 °C for 30 s and 72 °C 90 s (Acquiring Green). The PCR products were purified using QIAquick Gel Extraction kit (Qiagen Valencia, CA, USA) and analysed by dideoxy Sanger sequencing. Sequencing was carried on an ABI Prism 377 automatic sequencer (Applied Biosystems, Milan, Italy), using the ABI Prism Dye Terminator cycle sequencing Ready Reaction kit. The sequences were aligned using BioEdit sequence alignment editor 7.1.3.0.

Given the low quality of DNA extracted from FFPE specimens and the low viral load in tumour biopsies, we succeeded to obtain a complete sequence in only two porocarcinoma and two Merkel cell carcinoma samples.

As reported in Table 2, one mutation leading to a truncating form of the viral LT-Ag (presence of stop codon) was found in one MCC sample (MCC 2) but not in MCC 1. No truncating mutations were detected in the sequences obtained from two porocarcinomas. Few synonymous and nonsynonymous mutations in the viral helicase and OBD (origin binding domain) were detected in porocarcinoma specimens indicating a low genetic divergence of the virus identified in this tumour.

**Table 2:** Mutations detected in LT-Antigen coding sequences

Sample	Synonymous mutations	Domain	Nonsynonymous mutations	Domain	Stop mutations	Domain
SS1						
SS2	2393 T→ C (Ile)	helicase	1102 G→A (Arg→Lys)	Rb		
MCC1	2210 G→A(Arg)	helicase	253 G→T (Ala→Ser)	exon 1		
MCC2			253 G→T (Ala→Ser) 1666 A→G (Glu→Gly) 1766 G→C (Arg→Thr) 1768 T→C (Val→Ala) 1998 T→A (Phe→Ile) 2467 C→G (Ala→Gly)	exon 1 OBD OBD OBD helicase helicase	1504 C→G (Ser→Stop)	Rb-OBD
Poro1	2393 T→C (Ile)	helicase				
Poro2	1265 C→T (Phe) 2327 G→A (Glu)	Rb helicase	2453 G→T (Leu→Phe)	helicase		

SS1, SS2: salivary swabs; MCC1, MCC2: Merkel cell carcinomas; Poro1, Poro2: porocarcinomas; RB: Rb binding domain; Base pair designation correspond to the prototype HF (accession number: JF813003). SS1 sequence was used as reference.

However, the analysis of electropherograms of Sanger sequencing revealed that both porocarcinoma samples and MCC1 biopsy contained only wild type MCPyV while coexistence of wild type and mutated strain prevalent containing stop codon was observed in MCC2 specimen (Fig. 1).

**Fig. 1:**
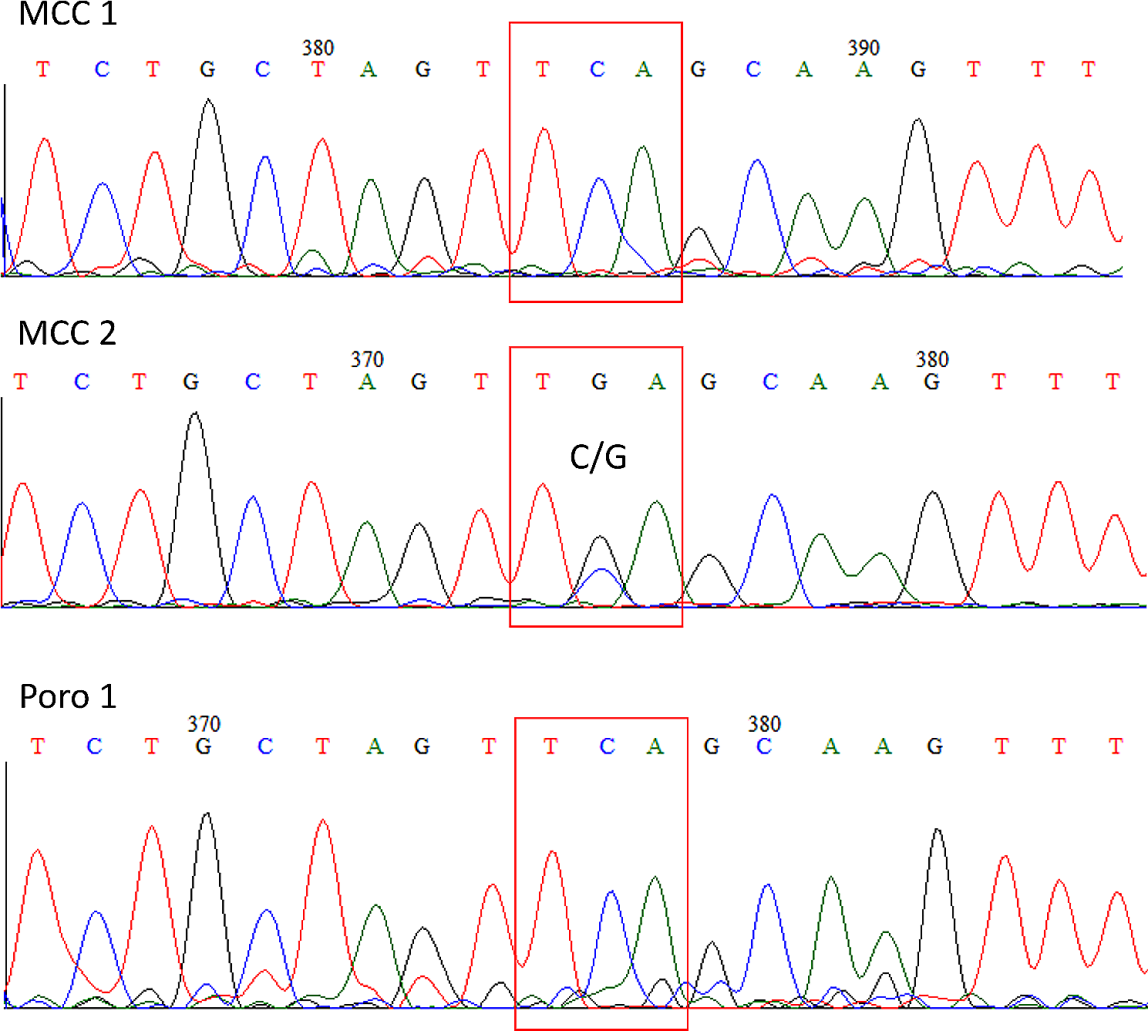
PCR amplification of LT encoding MCPyV sequences followed by Sanger sequencing

MCPyV is considered a component of normal skin flora, commonly present at low levels on the skin of healthy persons. In the absence of immunosurveillance, MCPyV can reactivate and, in a limited number of cases, can contribute to MCC pathogenesis.

Two independent genetic events, i.e., genomic integration and mutation/deletion of the MCPyV genome resulting in an encoded truncated LT, are virtually constantly observed in MCC tumors [13]. However, recently it has been demonstrated that, in some cases, both mutated and wild type LT sequences can coexist in MCC tissue [14]. In these cases, Sanger sequencing method could fail to detect the less prevalent or integrated sequence. To demonstrate the presence of truncating LT sequence in MCC more sensitive method, such as massive parallel sequencing, should be used. This method is also able detect integration events and nonsense mutations. In fact, we cannot exclude that lack of nonsense mutation detection in one of our MCC controls could be due to a low sensitivity of sequencing method used in this study. Unfortunately, we had not enough material to make immunohistochemical staining which could clarify whether T antigen is expressed. A high prevalence of MCPyV DNA in porocarcinoma compared to healthy controls (68% vs. 30% *p* < 0.04), detected in our previous study [12], prompted us to hypothesize the possible association between viral infection and the pathogenesis of this tumor. The lack of truncating mutations in LT-Ag sequence does not seem to support such a hypothesis. However, given the low quality of DNA isolated from FFPE specimens, we succeeded to get a complete sequence in only two cases. An analysis of more sequences coding for LT-Ag is necessary to clarify the role, if any, of the virus in the pathogenesis of this type of carcinoma. For further larger studies, fresh frozen tissues, which contain high quality DNA, could represent a more appropriate type of samples. Moreover, we cannot exclude that the mechanism of MCPyV oncogenesis in porocarcinoma differs from that proposed for MCC and that it does not depend on the LT-Ag mutations/deletions [15].

## Data Availability

No datasets were generated or analysed during the current study.

## Abbreviations

BCC: Basal cell carcinoma
BD: Bowen’s disease
SS1, SS2: Salivary swabs
FFPE: Formalin fixed paraffin embedded
LT-Ag: T antigen
MCC: Merkel cell carcinoma
MCPyV: Merkel cell polyomavirus
OBD: Origin binding
Poro1, Poro2: Porocarcinomas
RB: Rb binding domain
SCC: Squamous cell carcinoma
